# Effect of Surface Properties of Chitosan‐Based Nanoparticles in the Skin‐Diffusion Rate

**DOI:** 10.1002/bip.70006

**Published:** 2025-02-17

**Authors:** Luciana Ramírez, David Corral, Itandehui Betanzo, Deyanira Rodarte, Kanchan Chauhan, Rafael Vazquez‐Duhalt

**Affiliations:** ^1^ School of Medicine, Centro de Estudios Universidad Xochicalco Ensenada Baja California Mexico; ^2^ Centro de Nanociencias y Nanotecnología, Universidad Nacional Autónoma de México Ensenada Baja California Mexico; ^3^ Laboratory of Histology Universidad Autónoma de Baja California Ensenada Baja California Mexico

**Keywords:** chitosan nanoparticle, drug delivery, nanoparticle surface, skin diseases, transdermal administration

## Abstract

Skin diseases may cause rash, inflammation, itchiness, and other important skin changes, including dysplasia. Some skin conditions may be due to genetic and lifestyle factors and immune‐mediated factors. The current skin disease treatment can include oral medication, topical cream, or ointments. Nanotechnology is revolutionizing the drug delivery systems, increasing the time life of active therapeutic compounds and improving the treatment efficiency. This work hypothesizes that varying the surface properties of chitosan nanoparticles (Ch‐NPs) can modulate their diffusion through dermal tissue. Thus, Ch‐NPs were synthesized, and their surface was modified with polyethylene glycol, oxalic acid, and linoleic acid for transdermal therapy. The different Ch‐NPs were labeled with a fluorophore, and the dermal diffusion was measured on human skin by histological preparations and fluorescent microscopy. The surface properties of nanoparticles were shown to play an essential role in skin diffusion rate. Surface modification with a lipophilic moiety such as linoleic fatty acid showed a diffusion rate of 7.23 mm^2^/h in human full‐thickness abdominal flap, which is 2.7 times faster nanoparticle diffusion through dermal tissue when compared with the unmodified Ch‐NPs (2.92 mm^2^/h). The positive (zeta potential +27.5 mV) or negative (zeta potential −2.2 mV) surface charge does not affect the chitosan nanoparticle diffusion. Polyethylene glycol surface modification slightly improved the nanoparticle diffusion rate (3.63 mm^2^/h). Thus, modulating the nanoparticle surface properties can control the skin diffusion rate. The implications of this finding on dermic drug delivery are discussed.

## Graphical abstract

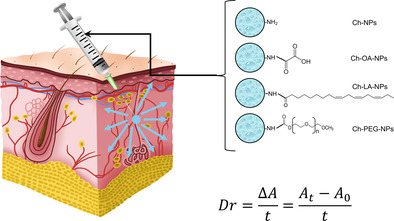

## 1 Introduction

Today, nanomedicine is improving a diversity of conventional treatments, optimizing the efficiency of current therapies by using different nanoparticle systems for drug delivery. Nanomedicine could be defined as the use of nanotechnology for diagnostic and therapeutic purposes [1], and includes the use of nanosized materials as carriers of therapeutic agents, including small molecules, lysosomes, proteins, peptides, and polymers to treat systemic diseases [2], or dermatological skin diseases [3].

There is a growing interest in developing minimally invasive methods and reducing systemic side effects caused by pharmacological treatments. An example is the transdermal delivery of drugs facilitated by nanocarriers [3]. Nanocarriers are doubtless, promising dermal, and transdermal drug delivery systems. Transdermal (for systemic effects) and topical (for local effects on the skin) delivery systems based on nanotechnology offer the following advantages: [4, 5, 6, 7] (i) A better therapeutic effect with reduced drug doses. (ii) Reduction of side effects at the systemic level or ineffective doses. (iii) Prevention of hepatic metabolism and interaction with the digestive system. (iv) Greater patient acceptance. In the case of dermatological diseases, topical administration is carried out only at the site of the affected tissue [6].

Nanocarriers have been fabricated from various materials, such as polymers, lipids, and metals. The unique properties of chitosan (an amino polysaccharide derived from chitin through a deacetylation process) make it an excellent biomaterial for various biomedical applications. One of the most notable properties of chitosan is that it does not cause severe inflammation or stimulation of the immune system. Chitosan preparations with various deacetylation degrees and molecular weights have low toxicity [8, 9]. In addition, some medical properties, such as anti‐ulcer [10], wound healing, and antibacterial properties [11, 12, 13] have been reported. In addition, due to their excellent biocompatibility, biodegradability, and adsorption properties, chitosan nanoparticles (Ch‐NPs) have been used as nanocarriers for drug delivery for oral, ophthalmic, intratumoral, transdermic, and systemic administrations [14, 15, 16].

The effect of the nanoparticle surface properties and the use of enhancers on skin penetration have been extensively studied [4, 7, 17]. However, there is scarce information on nanoparticle diffusion through the dermal tissue. The advancement of transdermal microneedle technology makes it essential to modulate the tissue diffusion of nanocarriers for therapeutic purposes [18, 19, 20].

In this work, Ch‐NPs have been chemically modified to obtain different surface properties, and their diffusion through the dermal tissue was evaluated.

## 2 Experimental Section

### 2.1 Materials

The deacetylated chitosan from crab shell, methoxy polyethylene glycol succinimidyl carboxymethyl ester, MW 5000 (M‐PEG‐SCM), sodium tripolyphosphate, *N‐*hydroxysuccinimide (NHS), oxalic acid, linoleic acid, and solvents were purchased at Sigma‐Aldrich (St. Louis, MO). L‐Glutamine and 1‐ethyl‐3‐(dimethylaminopropyl) carbodiimide (EDC) were purchased from Merck Millipore (Darmstadt, Germany). Invitrogen Cyanine5 (Cy5) dye was acquired at Thermo Fisher Scientific (Waltham, MA, USA). Buffer salts were obtained from J.T. Baker Chemicals (Mexico City).

The full‐thickness skin of the human abdominal was obtained as surgical waste from a plastic procedure.

### 2.2 Ch‐NP Synthesis

The Ch‐NPs were prepared using a modified ionic gelation method as previously described [21, 22], with some modifications. Chitosan (2.5% w/v) was dissolved in 2% glacial acetic acid in an aqueous solution. After complete dissolution, the chitosan solution was adjusted to pH 4.5 and stirred overnight at room temperature. Then, the solution was centrifuged for 30 min at 8000 rpm, and the supernatant was recovered and subsequently filtered through a funnel glass filter.

Several batches were prepared as described below, and the final products were pooled; 5 mL of fresh chitosan solution was placed under magnetic stirring, and 200 μL of Cy5 (3 mg/mL) was added to the stirring chitosan solution to obtain fluorescently labeled chitosan, which was kept protected from light and under darkness. The ionic gelation to form Ch‐NPs was obtained by adding 1 mL of sodium tripolyphosphate (0.025%) dropped at 0.09 mm/s with an automated injection‐controlled drip system (Poseidon software drop system) through a 3 mL syringe (8.66 mm i.d.) and stirred for 1 h. The nanoparticle preparation was crosslinked by adding 100 μL of glutaraldehyde (2.5%) dropped at 0.09 mm/s through a 1 mL syringe (4.6 mm i.d.) and left stirred for 1 h. The non‐reacted aldehyde groups were neutralized by adding 1 mL of 0.2‐mM glutamine dropped at 0.09 mm/s through a 3‐mL syringe (8.66 mm i.d.) and stirred for 30 min at room temperature. Finally, the chitosan nanoparticle suspension was centrifuged at 3500 rpm for 30 min at 4°C. The supernatant was recovered, and the pellet containing big debris was discarded. The supernatant was then centrifuged at 11,000 rpm, 1 h, 4°C. The Ch‐NPs pellet was resuspended in 1 mL of 50‐mM sodium phosphate buffer, pH 6.

### 2.3 Ch‐NP Modification

Four different nanoparticle preparations were obtained by chemical modification of the Ch‐NPs. Unmodified nanoparticles (Ch‐NPs) as positively charged surface, oxalic acid‐modified nanoparticles (Ch‐OA‐NPs) as negatively charged nanoparticles, linoleic acid‐modified nanoparticles (Ch‐LA‐NPs) as hydrophobic nanoparticles, and polyethylene glycol‐modified nanoparticles (Ch‐PEG‐NPs) as amphipathic nanoparticles.

Oxalic acid modification was carried out by diluting 2 mL of Ch‐NPs suspension with 2 mL of 50‐mM phosphate buffer pH 6.8. Then, 115 mg of NHS (*N*‐hydroxysuccinimide) and 96 mg of EDC (carbodiimide) were added. Finally, 58 mg of oxalic acid was added, and the mixture was left to react in agitation for 2 h. On the other hand, polyethylene glycol modification was performed by adding 635 mg polyethylene glycol (M‐PEG‐SCM) to diluted Ch‐NPs suspension and stirring for 2 h. For linoleic acid modification, 1.2 mL of absolute isopropanol was mixed with 2.8 mL of MiliQ water, and 80 μL of linoleic acid was added and mixed for 30 min. Then, 2 mL of diluted Ch‐NPs suspension was added, followed by 115 mg of NHS (N‐hydroxysuccinimide) and 96 mg of EDC (carbodiimide). The reaction mixture was stirred for 2 h. All preparations were dialyzed against 50‐mM phosphate buffer pH 6.8 to eliminate unreacted material and kept under darkness.

### 2.4 Nanoparticle Characterization

The hydrodynamic size of nanoparticles was estimated and analyzed by dispersion light scattering (DLS) using a Zetasizer nano ZS90 instrument (Malvern, UK). The effective surface charge was estimated as Z potential in a buffer of pH 6.8. The morphology of the nanoparticles was observed and evaluated by transmission electron microscopy (TEM) in a JEOL ARM‐200F with spherical aberration corrector, operated at 200 keV, and equipped with an EDS Oxford AZTecTEM detector. All measurements were carried out at least in triplicate (*n* = 3).

### 2.5 Skin Tissue Preparation and Nanoparticle Exposure

A full‐thickness abdominal flap was obtained as surgical waste from a plastic procedure (containing subcutaneous fatty tissue). The sample was collected immediately after the surgery, deposited on a sterile disposable surgical drape soaked with PBS buffer, and transported into a container with gel ice to the laboratory. The abdominal tissue was carefully cleaned with Milli Q water, and a clean internal section of 10 cm × 10 cm was dissected. Then, tissue pieces of 1 cm^2^ were obtained, which were used for the different treatments.

Nanoparticle suspensions (10 μL) were injected 1 mm deep (to be deposited in the dermis layer) with an insulin syringe with a 6 mm needle (Gauge 33). The different treatments were incubated at 37°C for 0, 1, and 4 h.

### 2.6 Histological Preparations

Each procedure was followed by an exposure time of 0, 1, and 4 h before performing cryo‐histological sample preparation. After exposure, cryomedium (Tissue Freezing Medium, Leica Biosystems Richmond Inc., Richmond, IL, USA) was added to the skin tissue. Vertical 8‐ and 10 μm sections were prepared using a cryotome (Microm Cryo‐Star HM 560, MICROM International GmbH, Walldorf, Germany) and transferred onto microscope slides. An inverse transmission microscope (Olympus IX 50, Olympus K. K., Shinjuku, Tokyo, Japan) was applied to check the quality of the histological preparations.

### 2.7 Fluorescence Microscopy

Fluorescence in the samples from the different treatments and incubation times was observed in an LS720 Microscope Lumascope (Etaluma Inc., San Diego, CA) equipped with a red filter (excitation 580–598 nm, emission 612–680 nm). The images were then analyzed by using the software Image J.

## 3 Results and Discussion

**FIGURE 1:**
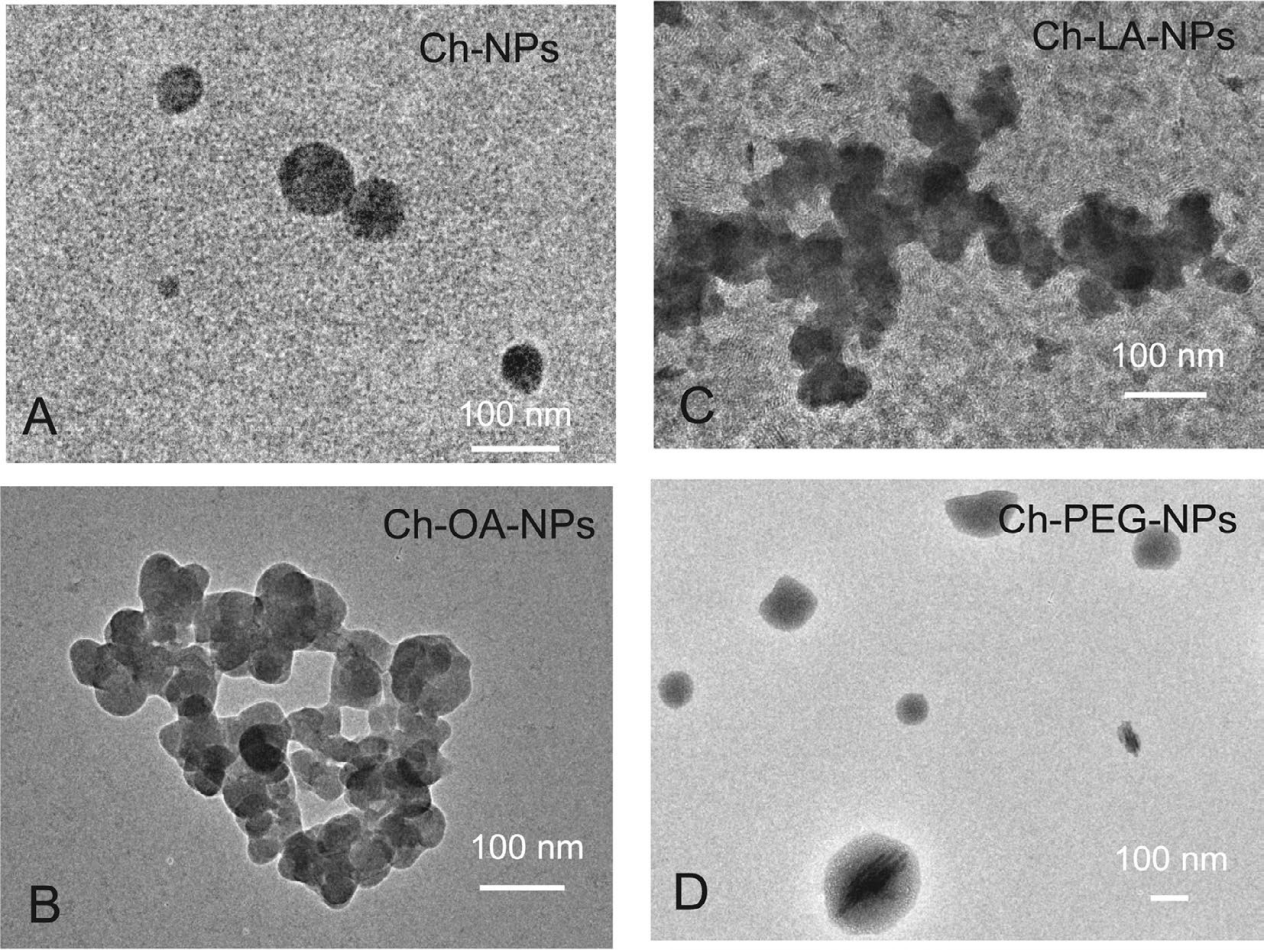
Images from transmission electron microscopy (TEM) of the surface unmodified and modified chitosan nanoparticles. (A) Unmodified chitosan nanoparticles. (B) Oxalic acid‐modified chitosan nanoparticles. (C) Linolenic acid‐modified chitosan nanoparticles. (D) Polyethylene glycol‐modified chitosan nanoparticles.

Ch‐NPs were produced using the ionic gelation technique to obtain nanocarriers for transdermal treatments. The shape and sizes were determined by TEM and dynamic light scattering (DLS), respectively. Quasispherical nanoparticles were constantly obtained (Figure 1A) with diameters ranging from 136 to 284 nm, an average hydrodynamic diameter of 242 nm (Table 1), and a positively charged surface with a zeta potential of 27.5 mV.

**TABLE 1:** Hydrodynamic diameter and zeta potential of chitosan nanoparticles after different functionalizations determined by dispersion light scattering (DLS).

Preparation	Hydrodynamic diameter (nm)	Zeta potential (mV)
Ch‐NPs	242 ± 60	27.5 ± 4.1
Ch‐OA‐NPs	231 ± 44	−2.2 ± 0.2
Ch‐LA‐NPs	361 ± 50	−4.1 ± 0.1
Ch‐PEG‐NPs	322 ± 52	−9.9 ± 1.0

The differences between nanoparticle diameter measured by TEM and hydrodynamic diameter measured by DLS are due to measurement technique. Hydrodynamic size indicates how the particle behaves in a fluid, in which the translational diffusion coefficient will depend not only on the size of the particle “core” but also on any surface structure, as well as the concentration and type of ions in the medium. The following sections discuss factors that affect particles' diffusion speed. On the other hand, TEM probes the electron‐rich part of the particle, and only the inner core will be seen, and the result obtained will be smaller.

In addition to surface charge and shape, nanoparticle size is an important parameter that affects biodistribution, kinetic release, and cell internalization. Smaller nanoparticles facilitate cell internalization, stability, and retention. This is the case for Ch‐NPs [23, 24], and it also seems to be the case for tissue diffusion [25, 26]. The size of Ch‐NPs obtained by ionic gelation could be modulated by both chitosan‐TPP proportions [27, 28] and the salt concentration in the synthesis medium [29].

Ch‐NPs have been studied for drug dermal delivery [30, 31], and chitosan coating has improved the skin permeation of gold nanoparticles [32] and lipid nanoparticles [33]. It has been proposed that a relatively stable, positively charged colloidal dispersion system facilitates the deep permeation in the *stratum corneum*.

Skin is the largest organ in the human body, and accessibility makes it an attractive port for drug administration. Medical dermic treatments have been used since early human times [7]. Topical formulations are now widespread, especially transdermal drug delivery, but have yet to reach their full potential. Nanoparticle skin penetration has been largely studied [4, 17, 34], even the effect of enhancers on the nanoparticle skin penetration rate [7, 35, 36]. On the other hand, nanoparticle tissue diffusion has been studied in tumor tissue [37, 38], eyes [39, 40], in simulated model materials [41, 42], and in silico [43, 44]. Nevertheless, despite the increase in dermal treatments, there is scarce information on nanoparticle diffusion through the skin tissues.

**FIGURE 2:**
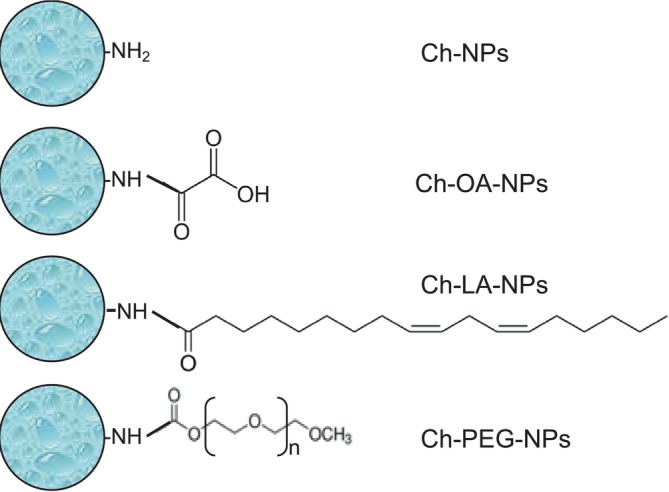
Schema of the different modifications on the surface of chitosan nanoparticles.

To study the effect of the surface properties of Ch‐NPs on the diffusion rate through the dermal tissue, Ch‐NPs were functionalized with different molecules (Figure 2). To obtain a negatively charged surface, Ch‐NPs were functionalized with oxalic acid by carbodiimide and N‐hydroxysuccinimide mediated reaction. In this modification, the free amino groups of glucosamine monomers of chitosan were covalently bonded to one of the carboxylic moieties of oxalic acid, exposing the other carboxylic group to the solvent. As expected, the zeta potential shown by Ch‐OA‐NPs was negative, with a value of −2.2 mV. Nevertheless, this value is not high enough to avoid nanoparticle aggregation, as shown in Figure 1B. The Ch‐OA‐NPs, after sonication, showed no size difference with the unfunctionalized nanoparticles with an average hydrodynamic diameter of 231 nm (Table 1).

Lipophilic Ch‐NPs were synthesized by covalently bonding linoleic acid molecules to the free amino groups of chitosan (Ch‐LA‐NPs). This preparation was also prone to aggregation due to their hydrophobicity (Figure 1C). The nanoparticles were disaggregated by ultrasonication, and then the sizes obtained by DLS (Table 1) showed an average hydrodynamic diameter of 361 nm. The TEM images clearly showed that the aggregates are formed by individual nanoparticles of around 300 nm.

Finally, an amphiphilic molecule, such as polyethylene glycol (PEG), was used to functionalize the chitosan nanoparticle's surface. The Ch‐PEG‐NPs showed a negatively charged surface and average hydrodynamic diameters of 322 nm, which is significantly bigger than the unmodified nanoparticles. This preparation is easily dispersed by sonication showing quasispherical nanoparticles (Figure 1D).

**FIGURE 3:**
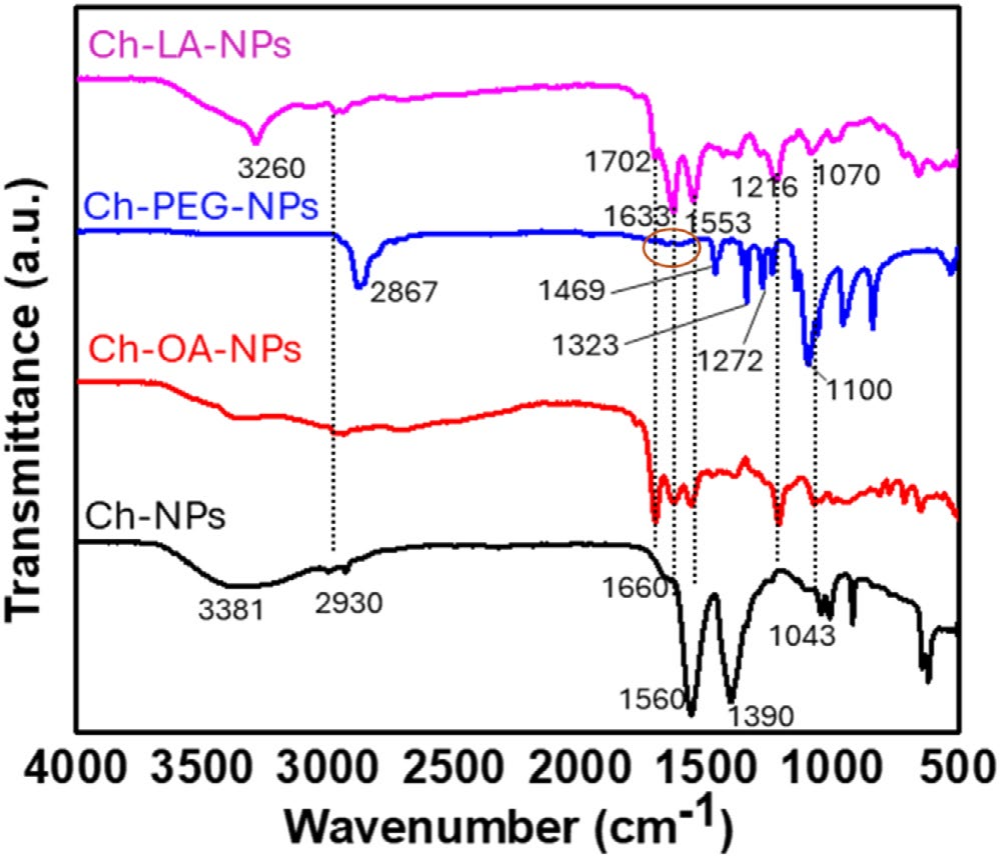
FTIR spectra of chitosan nanoparticles (Ch‐NPs), oxalic acid functionalized nanoparticles (Ch‐OA‐NPs), PEG‐SCM functionalized nanoparticles (Ch‐PEG‐NPs), linoleic acid functionalized nanoparticles (Ch‐LA‐NPs).

The FTIR spectra of Ch‐NPs, Ch‐PEG‐NPs, Ch‐OA‐NPs, and Ch‐LA‐NPs were analyzed and are compared in Figure 3 to illustrate the chemical modifications. The spectrum of Ch‐NPs exhibited characteristic bands, including a broad peak at 3381 cm^−1^ corresponding to NH₂ and OH stretching vibrations. The peaks at ~2900 cm^−1^ were assigned to the C‐H stretching vibrations of ‐CH₂ groups in chitosan, while bands at 1660 and 1560 cm^−1^ arose from interactions between the phosphate groups of TPP and amino groups of chitosan. A strong band at 1390 cm^−1^ was attributed to CH₂ wagging, and the P=O stretching vibration appeared at 1043 cm^−1^, consistent with previous findings [45].

In the spectrum of Ch‐PEG‐NPs, a significant reduction in the intensity of NH₂ and OH stretching bands was observed, likely due to hydrogen bonding between the hydroxyl and amine groups of chitosan and the hydroxyl groups of PEG [46]. Additionally, weak amide I (C=O stretching) and amide II (N‐H bending) bands, as highlighted in the spectrum of Ch‐PEG‐NPs were identified at ~1660 and ~1570 cm^−1^, respectively. The P=O stretching signal merged with the C‐O stretching vibration of PEG at ~1100 cm^−1^, reflecting the dominant presence of PEG. This suggests that PEG forms a surface layer over the Ch‐NPs and since FTIR detects surface chemical bonds more readily, the signals from the PEG groups overshadowed the chitosan core. Similar spectral changes were also observed earlier after the pegylation of Ch‐NPs [47].

Functionalization with linoleic acid and oxalic acid revealed distinct features. In both Ch‐LA‐NPs and Ch‐OA‐NPs, amide I and amide II bands at ~1630 and 1530 cm^−1^ confirmed covalent conjugation via amide bond formation [48]. The signal from the alkene group of linoleic acid in Ch‐LA‐NPs merged with OH and NH stretching vibrations, appearing as a broad peak at 3260 cm^−1^. In Ch‐OA‐NPs, the additional carboxyl group of oxalic acid enhanced the intensity of the carbonyl vibration at ~1700 cm^−1^, providing further evidence of surface modification. These spectral changes collectively confirm the successful functionalization of Ch‐NPs with PEG, linoleic acid, and oxalic acid.

**FIGURE 4:**
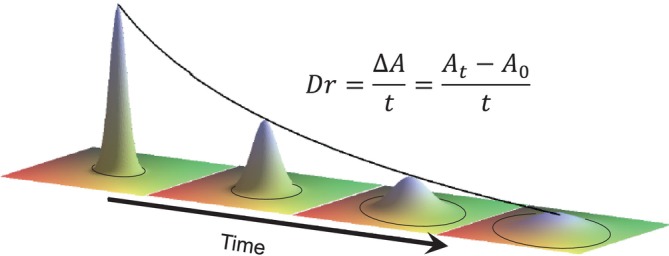
Schematic representation of the diffusion of fluorescent nanoparticles through the dermal tissue. The equation used for the estimation of diffusion rate is shown, where *Dr* is the diffusion rate in mm^2^/h; *A*_*t*_ is the fluorescence area in mm^2^ after 1‐h incubation; and *A*_0_ is the fluorescence area at time 0.

**FIGURE 5:**
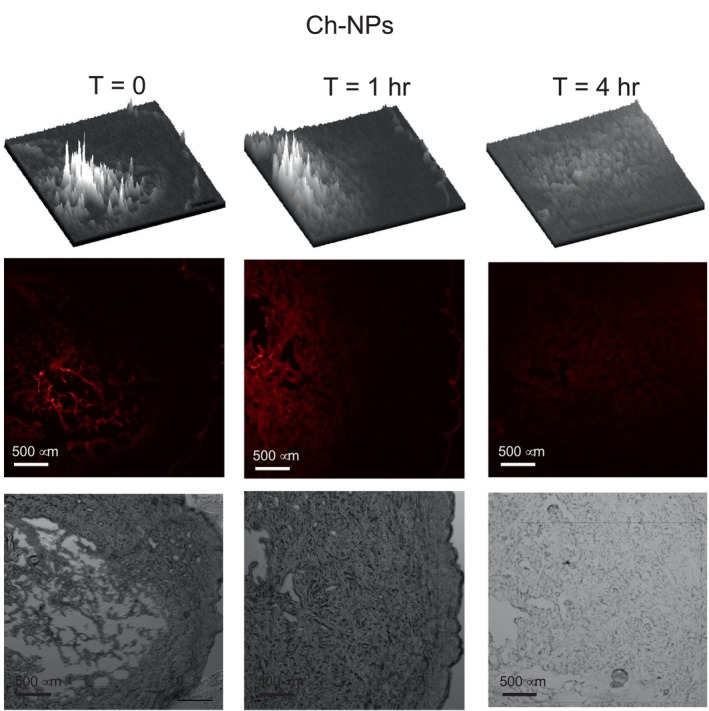
Images from fluorescence and bright field microscopy at different times of the dermal diffusion of unmodified chitosan nanoparticles (Ch‐NPs). At the top of each time is the 3D representation of the fluorescence intensity throughout the field view.

**FIGURE 6:**
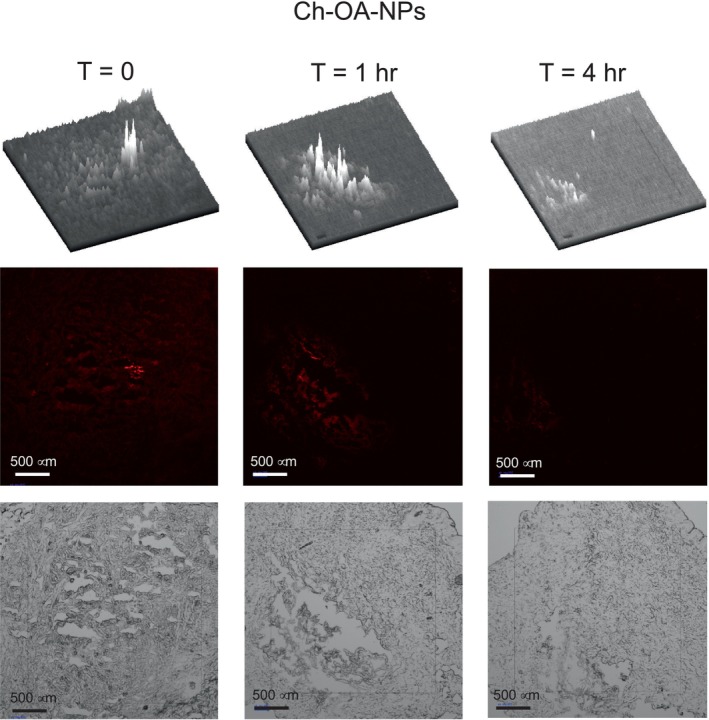
Images from fluorescence and bright field microscopy at different times of the dermal diffusion of oxalic acid‐modified chitosan nanoparticles (Ch‐OA‐NPs). In the top of each time is the 3D representation of the fluorescence intensity throughout the field view.

**FIGURE 7:**
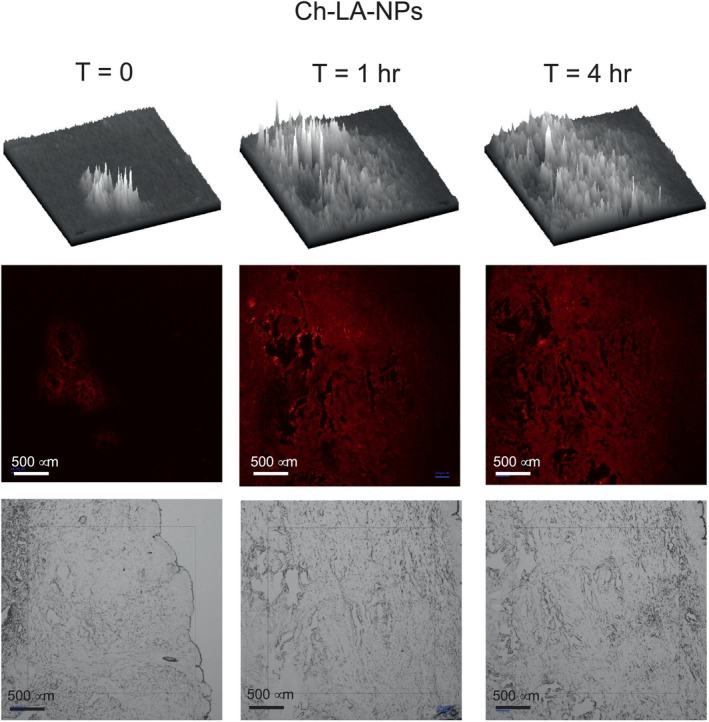
Images from fluorescence and bright field microscopy at different times of the dermal diffusion of linolenic acid‐modified chitosan nanoparticles (Ch‐LA‐NPs). In the top of each time is the 3D representation of the fluorescence intensity throughout the field view.

The nanoparticles were labeled with the red fluorophore (Cyanine 5 dye, Cy5) to follow the diffusion through the skin tissue. All four preparations were tested for diffusion inside human abdominal full‐thickness skin. Teen microliters of nanoparticle suspensions were injected 1 mm deep into the tissue and then incubated at 37°C for 0, 1, and 4 h. Vertical sections of 8 and 10 μm were prepared employing a cryotome, fixed, and observed in a fluorescence microscope. At time zero, the fluorescence is expected to be concentrated in a reduced tissue area (Figure 4). After incubation times, the fluorescence diffuses, covering a more extensive area. The diffusion continues during incubation, making the fluorescence less intense due to dilution. This diffusion is clearly observed in the 3D image at the top of Figures 4, 5, 6, 7.

The diffusion rate was estimated by using the following equation

$$\mathrm{Dr}=\frac{∆A}{t}=\frac{A_{t}-A_{0}}{t}$$

where *Dr* is the diffusion rate in mm^2^/h, *A*_*t*_ is the fluorescence area in mm^2^ after 1‐h incubation, and *A*_0_ is the fluorescence area at time 0.

**FIGURE 8:**
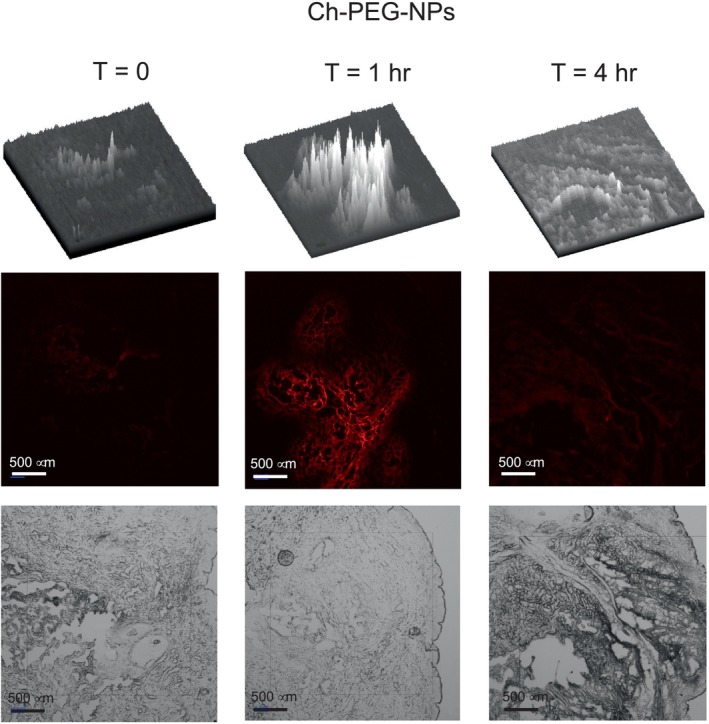
Images from fluorescence and bright field microscopy at different times of the dermal diffusion of polyethylene glycol‐modified chitosan nanoparticles (Ch‐PEG‐NPs). In the top of each time is the 3D representation of the fluorescence intensity throughout the field view.

Figures 5, 6, 7, 8 show the images of all four preparations at different incubation times. The estimated diffusion rates are shown in Table 2. Negatively charged nanoparticles (Ch‐OA‐NPs) showed a lower diffusion rate than the unmodified and positively charged nanoparticles (Ch‐NPs). Nanoparticles modified with PEG (Ch‐PEG‐NPs) showed a slightly faster diffusion rate than the unmodified ones. Finally, the diffusion rate of lipophilic nanoparticles (Ch‐LA‐NPs) was 2.5 times faster than the unmodified chitosan nanoparticles (Table 2).

**TABLE 2:** Estimated diffusion rates as the increase of the fluorescent area in the vertical histological preparations at the injection time and 1‐h after injection.

Preparation	Estimated diffusion rate (mm^2^/h)
Ch‐NPs	2.92 ± 0.93
Ch‐OA‐NPs	2.21 ± 0.41
Ch‐LA‐NPs	7.23 ± 0.58
Ch‐PEG‐NPs	3.63 ± 0.75

*Note:* The nanoparticles were labeled with Cy5 fluorophore and the histological preparations were observed in a fluorescent microscope with a red filter. The area was calculated using Image J software.

The possibility of modulating the diffusion rate of nanoparticles through the dermal tissue by surface modification has been demonstrated in this work (Table 2 and Figures 5, 6, 7, 8). No significant differences in the diffusion rate were found in the charged positively (Ch‐NPs) or negatively (Ch‐OA‐NPs). Nevertheless, amphiphilic (Ch‐PEG‐NPs), and especially hydrophobic (Ch‐LA‐NPs) chitosan nanoparticles, showed 1.6 and 2.5 times higher diffusion rates, respectively, than unmodified ones.

PEG surface modification is widely used to improve the properties of nanoparticles, including their active targeting, biocompatibility, mucus permeability, and pharmacokinetics. In addition, surface PEGylation avoids the aggregation of nanoparticles to increase their colloidal stability [49, 50]. Also, the surface PEG coating was shown to change the nanoparticle‐mediated skin drug delivery using polymeric nanoparticles [51], liposomes [52], and silica nanoparticles [53].

Lipophilic compounds, such as fatty acids, have been extensively studied as skin penetration enhancers [7, 36]. Kanikkannan et al. [54] and Vavrova et al. [55] reviewed the structure relationships of the aliphatic chains on skin penetration. From these works, the enhancers with unsaturated chains of 18–20 carbons seem to be the best for skin penetration [56]. Oleic acid is the most widely studied.

Dermatokinetics are defined as drug diffusion through various skin layers, and it is still one of the biggest challenges in developing topical products. Different in vitro, ex vivo, and in vivo techniques have been used for dermatokinetic studies [57]. In this work, we have used real human skin flaps from plastic surgical procedures to estimate the diffusion rate of the different nanoparticle preparations.

Transdermal treatment administration with microneedles has been introduced as a tool for physically enhancing drug‐loaded nanoparticle delivery. Microneedles arrays can pierce the underlying skin layers of interest with minimal invasiveness. Nanoparticle delivery by microneedles is thus performed by the generation of reversible microchannels [19, 20, 58].

## 4 Conclusions

Ch‐NPs are an attractive alternative for transdermal treatments. Ch‐NPs can carry different cargoes, including a diversity of drug molecules. The nanoparticle surface properties play an important role in nanoparticle diffusion through the dermal tissue. Ch‐NPs functionalized with a fatty acid showed faster diffusion through the dermal tissue than the unfunctionalized nanoparticles. Positive or negatively surface‐charged nanoparticles showed no significant difference in diffusion rate. Polyethylene glycol‐functionalized Ch‐NPs showed a slightly faster tissue diffusion than the unmodified and negatively charged nanoparticles. Thus, modulating the surface properties by chemical modification of free amino groups on the Ch‐NPs makes it possible to tune up their diffusion rate. The capacity to modulate the diffusion rate of nanocarriers through the dermal tissue is of potential interest in designing transdermal medical treatments. Future studies are needed to evaluate the effect of the surface properties of the nanoparticles on the release of different cargos and drugs.

## Author Contributions

L.R., K.C and D.C. performed the investigation and writing – original draft. I.B. and D.R. created the methodology. R.V.D. performed the conceptualization, formal analysis, writing – review and editing, and funding acquisition.

## Ethics Statement

The experimental protocol has been approved by the Bioethical Committee of the Center for Nanoscience and Nanotechnology, UNAM.

## Conflicts of Interest

The authors declare no conflicts of interest.

## Data Availability

The data that support the findings of this study are available on request from the corresponding author. The data are not publicly available due to privacy or ethical restrictions.

## References

[1] V. Wagner , A. Dullaart , A. Bock , and A. Zweck , “The Emerging Nanomedicine Landscape,” Nature Biotechnology 24 (2006): 1211. 10.1038/nbt1006-1211 17033654

[2] R. Petros and J. DeSimone , “Strategies in the Design of Nanoparticles for Therapeutic Applications,” Nature Reviews. Drug Discovery 9 (2010): 615. 20616808 10.1038/nrd2591

[3] S. Güngor and E. Kahraman , “Nanocarriers Mediated Cutaneous Drug Delivery,” European Journal of Pharmaceutical Sciences 158 (2021): 1. 10.1016/j.ejps.2020.105638 33176190

[4] A. Schroeter , T. Engelbrecht , H. H. Neubert , and A. Goebel , “New Nanosized Technologies for Dermal and Transdermal Drug Delivery. A Review,” Journal of Biomedical Nanotechnology 6 (2010): 511. 21329045 10.1166/jbn.2010.1149

[5] D. Papakostas , F. Rancan , W. Sterry , U. Blume‐Peytavi , and A. Vogt , “Nanoparticles in Dermatology,” Archives of Dermatological Research 303 (2011): 533–550. 21837474 10.1007/s00403-011-1163-7

[6] Z. Zhang , P. C. Tsai , T. Ramezanli , and B. B. Michniak‐Kohn , “Polymeric Nanoparticles‐Based Topical Delivery Systems for the Treatment of Dermatological Diseases,” Wiley Interdisciplinary Reviews. Nanomedicine and Nanobiotechnology 5 (2013): 205–218. 23386536 10.1002/wnan.1211 PMC3631287

[7] A. Kováčik , M. Kopečná , and K. Vávrová , “Permeation Enhancers in Transdermal Drug Delivery: Benefits and Limitations,” Expert Opinion on Drug Delivery 17 (2020): 145. 31910342 10.1080/17425247.2020.1713087

[8] M. Kong , X. G. Chen , K. Xing , and H. J. Park , “Antimicrobial Properties of Chitosan and Mode of Action: A State of the Art Review,” International Journal of Food Microbiology 144 (2010): 51–63. 20951455 10.1016/j.ijfoodmicro.2010.09.012

[9] L. Bugnicourt and C. Ladavière , “Interests of Chitosan Nanoparticles Ionically Cross‐Linked With Tripolyphosphate for Biomedical Applications,” Progress in Polymer Science 60 (2006): 1.

[10] A. Fini and I. Orienti , “The Role of Chitosan in Drug Delivery: Current and Potential Applications,” Journal of Health Care Technology 1 (2003): 43.

[11] E. I. Rabea , M. E. Badawy , C. V. Stevens , G. Smagghe , and W. Steurbaut , “Chitosan as Antimicrobial Agent: Applications and Mode of Action,” Biomacromolecules 4, no. 6 (2003): 1457–1465, 10.1021/bm034130m. 14606868

[12] S. Rashki , K. Asgarpour , H. Tarrahimofrad , et al., “Chitosan‐Based Nanoparticles Against Bacterial Infections,” Carbohydrate Polymers 251 (2019): 117108. 10.1016/j.carbpol.2020.117108 33142645

[13] S. Rashki , H. Safardoust‐Hojaghan , H. Mirzaei , et al., “Delivery LL37 by Chitosan Nanoparticles for Enhanced Antibacterial and Antibiofilm Efficacy,” Carbohydrate Polymers 291 (2022): 119634. 35698353 10.1016/j.carbpol.2022.119634

[14] S. Naskar , K. Koutsu , and S. Sharma , “Chitosan‐Based Nanoparticles as Drug Delivery Systems: A Review on Two Decades of Research,” Journal of Drug Targeting 27 (2019): 379. 30103626 10.1080/1061186X.2018.1512112

[15] K. Jafernik , A. Ładniak , E. Blicharska , et al., “Chitosan‐Based Nanoparticles as Effective Drug Delivery Systems—A Review,” Molecules 28 (2023): 1963. 36838951 10.3390/molecules28041963 PMC9959713

[16] J. Kurczewska , “Chitosan‐Based Nanoparticles With Optimized Parameters for Targeted Delivery of a Specific Anticancer Drug—A Comprehensive Review,” Pharmaceutics 15 (2023): 503. 36839824 10.3390/pharmaceutics15020503 PMC9961640

[17] B. Iqbal , J. Ali , and S. Baboota , “Recent Advances and Development in Epidermal and Dermal Drug Deposition Enhancement Technology,” International Journal of Dermatology 57 (2018): 646–660. 29430629 10.1111/ijd.13902

[18] T. S. Alster and P. M. Graham , “Microneedling: A Review and Practical Guide,” Dermatologic Surgery 44 (2018): 397–404. 28796657 10.1097/DSS.0000000000001248

[19] A. S. Al‐Japairai , S. Mahmood , H. S. Almurisi , et al., “Current Trends in Polymer Microneedle for Transdermal Drug Delivery,” International Journal of Pharmaceutics 587 (2020): 119673. 32739388 10.1016/j.ijpharm.2020.119673 PMC7392082

[20] S. Ruan , Y. Zhang , and N. Feng , “Microneedle‐Mediated Transdermal Nanodelivery Systems: A Review,” Biomaterials Science 9 (2021): 8065. 34752590 10.1039/d1bm01249e

[21] W. Fan , W. Yan , Z. Xu , and H. Ni , “Formation Mechanism of Monodisperse, Low Molecular Weight Chitosan Nanoparticles by Ionic Gelation Technique,” Colloids and Surfaces B: Biointerfaces 90 (2012): 21–27, 10.1016/j.colsurfb.2011.09.042. 22014934

[22] R. D. Koyani and R. Vazquez‐Duhalt , “Laccase Encapsulation in Chitosan Nanoparticles Enhances the Protein Stability Against Microbial Degradation,” Environmental Science and Pollution Research 23 (2016): 18850–18857. 27318485 10.1007/s11356-016-7072-8

[23] N. Duceppe and M. Tabrizian , “Advances in Using Chitosan‐Based Nanoparticles for In Vitro and In Vivo Drug and Gene Delivery,” Expert Opinion on Drug Delivery 7 (2010): 1191. 20836623 10.1517/17425247.2010.514604

[24] C. He , L. Yin , C. Tang , and C. Yin , “Size‐Dependent Absorption Mechanism of Polymeric Nanoparticles for Oral Delivery of Protein Drugs,” Biomaterials 33 (2012): 8569–8578. 22906606 10.1016/j.biomaterials.2012.07.063

[25] T. Banerjee , S. Mitra , A. K. Singh , R. K. Sharma , and A. Maitra , “Preparation, Characterization and Biodistribution of Ultrafine Chitosan Nanoparticles,” International Journal of Pharmaceutics 243 (2002): 93. 12176298 10.1016/s0378-5173(02)00267-3

[26] Z. He , J. L. Santos , H. Tian , et al., “Scalable Fabrication of Size‐Controlled Chitosan Nanoparticles for Oral Delivery of Insulin,” Biomaterials 130 (2017): 28–41. 28359018 10.1016/j.biomaterials.2017.03.028

[27] G. Thandapani , S. P. Prasad , P. N. Sudha , and A. Sukumaran , “Size Optimization and In Vitro Biocompatibility Studies of Chitosan Nanoparticles,” International Journal of Biological Macromolecules 104 (2017): 1794. 28807691 10.1016/j.ijbiomac.2017.08.057

[28] J. Antoniou , F. Liu , H. Majeed , J. Qi , W. Yokoyama , and F. Zhong , “Physicochemical and Morphological Properties of Size‐Controlled Chitosan–Tripolyphosphate Nanoparticles,” Colloids and Surfaces A: Physicochemical and Engineering Aspects 465 (2015): 137–146, 10.1016/j.colsurfa.2014.10.040.

[29] Y. Huang and Y. Lapitsky , “Monovalent Salt Enhances Colloidal Stability During the Formation of Chitosan/Tripolyphosphate Microgels,” Langmuir 27 (2011): 10392–10399. 21749043 10.1021/la201194a

[30] A. Hasanovic , M. Zehl , G. Reznicek , and C. Valenta , “Chitosan‐Tripolyphosphate Nanoparticles as a Possible Skin Drug Delivery System for Aciclovir With Enhanced Stability,” Journal of Pharmacy and Pharmacology 61 (2009): 1609–1616. 19958582 10.1211/jpp/61.12.0004

[31] Q. Ta , J. Ting , S. Harwood , et al., “Chitosan Nanoparticles for Enhancing Drugs and Cosmetic Components Penetration Through the Skin,” European Journal of Pharmaceutical Sciences 160 (2021): 105765. 33607243 10.1016/j.ejps.2021.105765

[32] N. Jaber , F. Al‐Akayleh , R. A. Abdel‐Rahem , and M. Al‐Remawi , “Characterization Ex Vivo Skin Permeation and Pharmacological Studies of Ibuprofen Lysinate‐Chitosan‐Gold Nanoparticles,” Journal of Drug Delivery Science and Technology 62 (2021): 102399.

[33] L. Marquez‐Andrade , L. Dantas , A. Krawczyk‐Santos , et al., “Improved Tacrolimus Skin Permeation by Co‐Encapsulation With Clobetasol in Lipid Nanoparticles: Study of Drug Effects in Lipid Matrix by Electron Paramagnetic Resonance,” European Journal of Pharmaceutics and Biopharmaceutics 119 (2017): 142. 28627400 10.1016/j.ejpb.2017.06.014

[34] F. Sabbagh and B. S. Kim , “Recent Advances in Polymeric Transdermal Drug Delivery Systems,” Journal of Controlled Release 341 (2022): 132–146. 34813879 10.1016/j.jconrel.2021.11.025

[35] P. Lee , N. Ahmad , R. Langer , S. Mitragotri , and P. Shastri , “Evaluation of Chemical Enhancers in the Transdermal Delivery of Lidocaine,” International Journal of Pharmaceutics 308 (2006): 33. 16321488 10.1016/j.ijpharm.2005.10.027

[36] H. Marwah , T. Garg , A. K. Goyal , and G. Rath , “Permeation Enhancer Strategies in Transdermal Drug Delivery,” Drug Delivery 23 (2014): 564. 25006687 10.3109/10717544.2014.935532

[37] V. Raeesi and W. Chan , “Improving Nanoparticle Diffusion Through Tumor Collagen Matrix by Photo‐Thermal Gold Nanorods,” Nanoscale 8 (2016): 12524. 26822539 10.1039/c5nr08463f

[38] X. He , Y. Yang , Y. Han , et al., “Extracellular Matrix Physical Properties Govern the Diffusion of Nanoparticles in Tumor Microenvironment,” Proceedings of the National Academy of Sciences of the United States of America 120, no. 1 (2023): e2209260120, 10.1073/pnas.2209260120. 36574668 PMC9910605

[39] Q. Xu , N. J. Boylan , J. S. Suk , et al., “Nanoparticle Diffusion in, and Microrheology of, the Bovine Vitreous Ex Vivo,” Journal of Controlled Release 167 (2013): 76–84. 23369761 10.1016/j.jconrel.2013.01.018 PMC3693951

[40] S. Swetledge , J. P. Jung , R. Carter , and C. Sabliov , “Distribution of Polymeric Nanoparticles in the Eye: Implications in Ocular Disease Therapy,” Journal of Nanbiotechnology 19 (2021): 10. 10.1186/s12951-020-00745-9 PMC7789499 33413421

[41] L. Xue , O. Borodin , and G. D. Smith , “Modeling of Enhanced Penetrant Diffusion in Nanoparticle‐Polymer Composite Membranes,” Journal of Membrane Science 286 (2006): 293–300.

[42] M. Sun , J. Lee , Y. Chen , and K. Hoshino , “Studies of Nanoparticle Delivery With In Vitro Bio‐Engineered Microtissues,” Bioactive Materials 5 (2020): 924–937. 32637755 10.1016/j.bioactmat.2020.06.016 PMC7330434

[43] P. A. Wijeratne and V. Vavourakis , “A Quantitative In Silico Platform for Simulating Cytotoxic and Nanoparticle Drug Delivery to Solid Tumours,” Interface Focus 9, no. 3 (2019): 20180063, 10.1098/rsfs.2018.0063. 31065337 PMC6501342

[44] N. R. Stillman , M. Kovacevic , I. Balaz , and S. Hauert , “In Silico Modelling of Cancer Nanomedicine, Across Scales and Transport Barriers,” NPJ Computational Materials 6 (2020): 92.

[45] C. Lustriane , F. M. Dwivany , V. Suendo , M. Reza , and M. J. Plant , “Effect of Chitosan and Chitosan‐Nanoparticles on Post Harvest Quality of Banana Fruits,” Journal of Plant Biotechnology 45 (2018): 36–44.

[46] R. A. Masud , M. S. Islam , P. Haque , et al., “Preparation of Novel Chitosan/Poly (Ethylene Glycol)/ZnO Bionanocomposite for Wound Healing Application: Effect of Gentamicin Loading,” Materialia 12 (2020): 100785.

[47] M. N. Melo , F. M. Pereira , M. A. Rocha , et al., “Chitosan and Chitosan/PEG Nanoparticles Loaded With Indole‐3‐Carbinol: Characterization, Computational Study and Potential Effect on Human Bladder Cancer Cells,” Materials Science and Engineering: C 124 (2021): 112089, 10.1016/j.msec.2021.112089. 33947529

[48] V. Taghipour‐Sabzevar , T. Sharifi , S. Bagheri‐Khoulenjani , et al., “Targeted Delivery of a Short Antimicrobial Peptide Against CD44‐Overexpressing Tumor Cells Using Hyaluronic Acid‐Coated Chitosan Nanoparticles: An In Vitro Study,” Journal of Nanoparticle Research 22 (2020): 99.

[49] J. S. Suk , Q. Xu , N. Kim , J. Hanes , and L. M. Ensign , “PEGylation as a Strategy for Improving Nanoparticle‐Based Drug and Gene Delivery,” Advanced Drug Delivery Reviews 99 (2016): 28–51. 26456916 10.1016/j.addr.2015.09.012 PMC4798869

[50] L. Shi , J. Zhang , M. Zhao , et al., “Effects of Polyethylene Glycol on the Surface of Nanoparticles for Targeted Drug Delivery,” Nanoscale 13 (2021): 10748–10764. 34132312 10.1039/d1nr02065j

[51] A. Lalloz , M. A. Bolzinger , S. Briançon , et al., “Subtle and Unexpected Role of PEG in Tuning the Penetration Mechanisms of PLA‐Based Nano‐Formulations Into Intact and Impaired Skin,” International Journal of Pharmaceutics 563 (2019): 79. 30825557 10.1016/j.ijpharm.2019.02.039

[52] W. Rangsimawong , P. Opanasopit , T. Rojanarata , and T. Ngawhirunpat , “Terpene‐Containing PEGylated Liposomes as Transdermal Carriers of a Hydrophilic Compound,” Biological & Pharmaceutical Bulletin 37 (2014): 1936. 25297807 10.1248/bpb.b14-00535

[53] J. H. Al Mahrooqi , V. V. Khutoryanskiy , and A. C. Williams , “Thiolated and PEGylated Silica Nanoparticle Delivery to Hair Follicles,” International Journal of Pharmaceutics 593 (2021): 120130. 33264642 10.1016/j.ijpharm.2020.120130

[54] N. Kanikkannan , K. Kandimalla , S. S. Lamba , and M. Singh , “Structure‐Activity Relationship of Chemical Penetration Enhan‐Cers in Transdermal Drug Delivery,” Current Medicinal Chemistry 7 (2000): 593–608. 10702628 10.2174/0929867003374840

[55] K. Vavrova , J. Zbytovska , and A. Hrabalek , “Amphiphilic Transdermal Permeation Enhancers: Structure‐Activity Relationships,” Current Medicinal Chemistry 12 (2005): 2273–2291. 16178785 10.2174/0929867054864822

[56] K. Morimoto , H. Tojima , T. Haruta , M. Suzuki , and M. Kakemi , “Enhancing Effects of Unsaturated Fatty Acids With Various Structures on the Permeation of Indomethacin Through Rat Skin,” Journal of Pharmacy and Pharmacology 48 (1996): 1133–1137. 8961160 10.1111/j.2042-7158.1996.tb03908.x

[57] P. Patel , S. Schmieder , and K. Krishnamurthy , “Research Techniques Made Simple: Drug Delivery Techniques, Part 2: Commonly Used Techniques to Assess Topical Drug Bioavailability,” Journal of Investigative Dermatology 136 (2016): 43. 10.1016/j.jid.2016.03.010 27107377

[58] X. Jiang , H. Zhao , and W. Li , “Microneedle‐Mediated Transdermal Delivery of Drug‐Carrying Nanoparticles,” Frontiers in Bioengineering and Biotechnology 10 (2022): 840395. 35223799 10.3389/fbioe.2022.840395 PMC8874791

